# Commentary: Zebrafish as a Model for Osteoporosis—An Approach to Accelerating Progress in Drug and Exercise-Based Treatment

**DOI:** 10.3390/ijerph192315866

**Published:** 2022-11-29

**Authors:** Natnaiel M. Dubale, Carolyn M. Kapron, Sarah L. West

**Affiliations:** 1 Department of Biology, Trent University, Peterborough, ON K9L 0G2, Canada; 2 Department of Kinesiology, Trent University, Peterborough, ON K9L 0G2, Canada; 3 Trent/Fleming School of Nursing, Trent University, Peterborough, ON K9L 0G2, Canada

**Keywords:** zebrafish, osteoporosis, animal model, bone, exercise, drug discovery

## Abstract

Osteoporosis (OP) is a degenerative disease characterized by reduced bone strength and increased fracture risk. As the global population continues to age, the prevalence and economic burden of osteoporosis can be expected to rise substantially, but there remain various gaps in the field of OP care. For instance, there is a lack of anti-fracture drugs with proven long-term efficacy. Likewise, though exercise remains widely recommended in OP prevention and management, data regarding the safety and efficacy for patients after vertebral fracture remain limited. This lack of evidence may be due to the cost and inherent difficulties associated with exercise-based OP research. Thus, the current research landscape highlights the need for novel research strategies that accelerate OP drug discovery and allow for the low-cost study of exercise interventions. Here, we outline an example of one strategy, the use of zebrafish, which has emerged as a potential model for the discovery of anti-osteoporosis therapeutics and study of exercise interventions. The strengths, limitations, and potential applications of zebrafish in OP research will be outlined.

Osteoporosis (OP) is a metabolic bone disease characterized by reduced bone strength, increased fracture risk [1,2], and fracture-associated increased mortality among patients [1]. With an estimated global prevalence of 18.3% [3], OP is not only a condition of significant public health concern, but one that produces a substantial economic burden [4]. As it is anticipated that the global population will continue to age [5], the prevalence, economic, and physiological impact of OP can be expected to rise substantially. OP management includes the use of pharmacotherapy (e.g., bisphosphonates), exercise prescription (e.g., resistance training), and/or dietary considerations (e.g., vitamin D_3_ and calcium supplementation) [6,7,8,9]. However, there are key gaps in the field of OP care that must be addressed to best respond to the needs of an aging global population. For instance, despite bisphosphonates being the primary drugs administered in OP treatment [6,7,8,10], there is limited evidence regarding their long-term efficacy [11], and their long-term use has been associated with certain rare adverse events (e.g., osteonecrosis of the jaw). While some encourage the use of ‘drug holidays’ or the temporary cessation of bisphosphonate therapy [12,13], a recent review suggests cessation of bisphosphonate therapy is associated with a 20–40% increase in the risk of new clinical fractures and a doubling of vertebral fracture risk [14]. There is currently a need for novel pharmaceuticals with proven anti-fracture efficacy after 5 years [15], and therapeutics that lack the rare adverse events associated with bisphosphonate use [15].

Exercise is widely recommended in the prevention and management of OP [6,7,8,16,17], but there are still gaps in our understanding of the impact of exercise in OP patients. Despite vertebral fractures being considered a hallmark of OP, there is insufficient evidence regarding the safety and efficacy of exercise interventions following such fractures in patients [18,19]. To address these gaps, randomized controlled exercise studies are needed [18,19]. One potential driver of the lack of data in this area may be the challenges and costs associated with conducting exercise-based OP research. As exercise both increases fracture risk (by potentially exposing bone to trauma when completed incorrectly) and reduces fracture risk (by improving bone quantity/quality), randomized controlled trials must be used to concretely establish the effects of exercise on bone [20]. Likewise, when assessing fracture risk as an endpoint in randomized controlled trials, far greater numbers of participants are required than for other endpoints [21], thereby raising the cost and complexity of such studies. One approach to address these research challenges is the development of robust, reliable animal models that allow for the translational study of interventions. As interventions based on such robust data are likely to find far greater success during randomized trials, such models may help spur a greater desire among researchers to undertake clinical OP studies. In the current commentary, we discuss one excellent strategy, the use of *Danio rerio* (zebrafish), which has emerged as a potential effective and low-cost model organism for the study of both drug- and exercise-based OP treatments [22,23,24,25].

There are numerous characteristics of the zebrafish that make this organism attractive for use in research. Zebrafish exhibit a short generation time of 2 to 4 months [26], which allows research to progress at a rapid pace and enables greater numbers of experiments to be conducted within a given timeframe [27,28]. Their high fecundity, with a single zebrafish female being capable of producing 200–300 eggs per week [29], can decrease the cost per assay [27]. Zebrafish can be maintained in a laboratory setting with relatively low running costs [30], and year-round breeding [31] ensures that zebrafish-based studies can proceed continuously rather than being limited to a set annual timeframe [27]. An additional strength of zebrafish as a model organism is that the zebrafish genome can be manipulated with ease. As zebrafish eggs are externally fertilized, embryos can be easily accessed at the one-cell stage for microinjection with gene-altering agents. Mutant zebrafish lines whose characteristics mimic human skeletal pathologies can be developed using currently available gene modification tools such as zinc-finger nucleases or the CRISPR/Cas9 system [32,33,34,35,36]. For instance, CRISPR/Cas9-mediated disruption of *itga10* or *itgbl1* has been shown to induce an osteoporosis-like phenotype in zebrafish [37].

Not only is zebrafish a convenient animal model to work with, another important and useful feature is the large amount of conservation of genetic sequences between humans and zebrafish: 71.4% of human protein-coding genes have a zebrafish orthologue [38]. This is accompanied by numerous similarities in bone physiology between zebrafish and humans, including similarities in bone cell types and ossification mechanisms [39]. Thus, in addition to genomic conservation, the parallels in physiology between zebrafish and humans further enhance the potential that results generated from zebrafish models are of translational value. Zebrafish also readily absorb compounds from their aquatic environment, therefore dissolved chemical agents can be used to induce abnormal phenotypes [40,41,42,43] as outlined in Figure 1. This approach has been employed to develop osteoporosis-like phenotypes in zebrafish, using chemicals such as glucocorticoids (e.g., prednisolone or dexamethasone) or ferric ammonium citrate [40,44,45,46]. Zebrafish are also well suited for use in high-throughput, whole-organism, phenotypic drug screens, designed to discover novel therapeutics [42,47]. Once developed, a key application of such models is that they can be used to screen for potential anti-osteoporotic drugs [44]. In a recent study, resveratrol, a plant polyphenol, was shown to significantly improve a glucocorticoid-induced osteoporosis-like phenotype in zebrafish, which suggests a potential for resveratrol to be used in OP prevention [48]. Screening candidate therapeutic molecules in zebrafish models for OP may be one effective strategy to accelerate progress in OP therapeutics research.

Another strength of the zebrafish model system is the ability to characterize skeletal phenotypes in vivo. As zebrafish embryos and larvae are optically transparent, fluorescent reporter lines (such as the *Tg(ola.sp7:nlsGFP)* mutant line) [49] can be used to visualize the zebrafish skeleton in real time. Fluorescent vital stains (such as calcein) [41,50] also provide the ability to conduct skeletal imaging in live zebrafish, and the use of transparent zebrafish of the *casper* strain enables such fluorescent imaging to be conducted in adult fish [51]. In the context of drug discovery, fluorescent imaging provides the ability to more rapidly and efficiently screen candidate molecules for their skeletal impacts and thus their potential as human anti-OP therapeutics [41,44]. Zebrafish bones can also be directly analyzed using various techniques such as whole-mount bone staining [52] and micro-computed tomography [53].

In addition to drug therapeutic studies, zebrafish may also be used to study the impact of exercise on bone [54], similar to zebrafish-based research on the pathways underlying vertebrate muscle hypertrophy and remodeling [25,55,56,57]. To assess bone quality, zebrafish may be euthanized, and their bones removed for external analysis [52]. Like humans, zebrafish exhibit age-associated declines in exercise performance and trainability [24]. Such a characteristic is highly advantageous, providing the potential for exercise interventions studied in aged zebrafish to be of translational value when developing programs for aging humans. Various cost-effective, benchtop apparatuses to study exercise in zebrafish have been developed [58,59,60]. Such systems include the ‘French press’, a high-throughput approach that entails the use of a coffee plunger and magnetic stirrer to produce water currents, enabling zebrafish to be exercised and trained [58]. Exercise-induced changes to bone can also be studied using a controlled swimming program such as the one developed by Suniaga et al. Zebrafish were placed to swim against laminar currents in swimming chambers, and zebrafish displayed bone adaptation in response to musculoskeletal exercise [54]. While exercise interventions for OP have yet to be studied in zebrafish [54], research conducted in a zebrafish model for Duchenne muscular dystrophy indicates that certain forms of exercise may be beneficial to patients with this condition [61]. Thus, the study of exercise-based interventions for OP in zebrafish models for OP is a promising area for future research [54].

Like all model organisms and research modalities, zebrafish-based research has various challenges. While institutions such as the Zebrafish International Resource Center (ZIRC) [62] can provide a host of mutant zebrafish lines to researchers, in Canada it can be difficult to import zebrafish lines [63]. Studies of zebrafish proteins may be limited by a current lack of reliable zebrafish-specific antibodies. Moreover, numerous physiological differences do exist between humans and zebrafish [39] which may complicate the translation of the findings of certain studies in zebrafish to humans. For instance, zebrafish (unlike humans) do not possess bone marrow, with definitive hematopoiesis occurring instead in the kidney marrow [39,64]. In addition, zebrafish have undergone genome-wide duplication [65] (such as with insulin-like growth factor) [66], which potentially limits applicability of this model’s results to other vertebrates. In addition, while research has supported the successful use of zebrafish as an exercise model [25], there are certainly exercise-associated research limitations that exist. For example, there is an inability to examine different types of exercise (such as weight-bearing activity, or resistance training) in addition to sedentary behaviour, which may independently impact outcomes in chronic diseases such as bone health in OP. Moreover, it is clear that the aquatic environment of the zebrafish is fundamentally different from that of humans [54]; another limiting consideration. Despite these limitations, based on our discussion above, we still suggest that zebrafish is an important model for scientists who are interested in advancing the area of OP intervention research.

In summary, OP remains a condition of substantial public health concern and there is a need for novel therapeutics. Research approaches that facilitate anti-osteoporosis drug discovery and the study of exercise interventions are therefore needed. In the current commentary, we outline an example of one excellent animal model, zebrafish, that can be used to accelerate research on OP pharmacotherapy and exercise-based treatment. Additionally, in the current research environment (i.e., the continued COVID-19 era), challenges with implementing clinical research in human participants beyond those discussed in the above commentary exist (such as physical distancing requirements, willingness of participants, etc.). This further supports the need for development of a robust clinical research program that uses effective non-human models to help inform clinical outcomes. In conclusion, the use of zebrafish as a model for OP is one example of a strategy that may aid in accelerating progress in OP care, thereby meeting the needs of an aging global population.

## Figures and Tables

**Figure 1:**
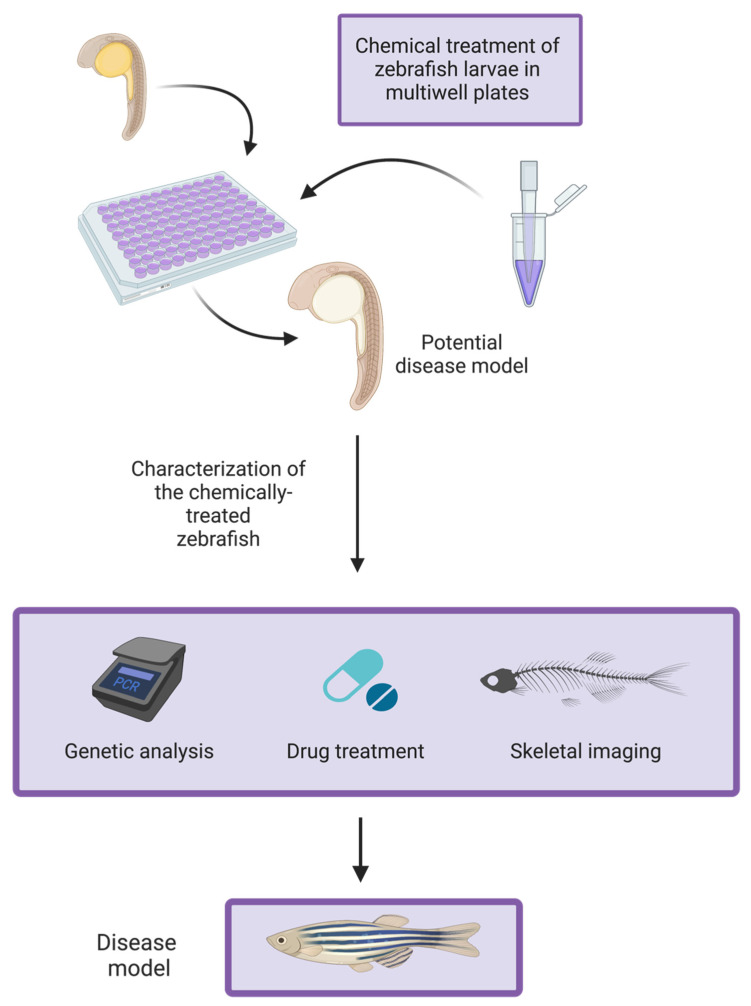
Schematic representation of a protocol to develop a zebrafish model for secondary OP using chemical treatments. Zebrafish larvae are placed in multiwell plates containing a dissolved chemical agent (e.g., glucocorticoids). The phenotype of the chemically-treated zebrafish is then characterized using a variety of approaches, including genetic analysis (e.g., via quantitative reverse transcription-polymerase chain reaction), skeletal imaging and drug treatments. Treatment of a candidate disease model with a drug designed to treat said condition in humans allows for the clinical relevance of the model to be evaluated. Information obtained from Barrett et al. (2006) [40], Huang et al. (2018) [44] and Zhang et al. (2018) [46]. Created with BioRender.com.

## References

[1] Brown J.P., Adachi J.D., Schemitsch E., Tarride J.E., Brown V., Bell A., Reiner M., Oliveira T., Motsepe-Ditshego P., Burke N. (2021). Mortality in older adults following a fragility fracture: Real-world retrospective matched-cohort study in Ontario. BMC Musculoskelet. Disord..

[2] Föger-Samwald U., Dovjak P., Azizi-Semrad U., Kerschan-Schindl K., Pietschmann P. (2020). Osteoporosis: Pathophysiology and therapeutic options. EXCLI J..

[3] Salari N., Ghasemi H., Mohammadi L., Rabieenia E., Shohaimi S., Mohammadi M. (2021). The global prevalence of osteoporosis in the world: A comprehensive systematic review and meta-analysis. J. Orthop. Surg. Res..

[4] Kanis J.A., Norton N., Harvey N.C., Jacobson T., Johansson H., Lorentzon M., McCloskey E.V., Willers C., Borgström F. (2021). SCOPE 2021: A new scorecard for osteoporosis in Europe. Arch. Osteoporos..

[5] United Nations Department of Economic and Social Affairs, Population Division World Population Prospects 2022: Summary of Results. UN DESA/POP/2022/TR/NO. 3. https://www.un.org/development/desa/pd/sites/www.un.org.development.desa.pd/files/wpp2022_summary_of_results.pdf.

[6] Papaioannou A., Morin S., Cheung A.M., Atkinson S., Brown J.P., Feldman S., Hanley D.A., Hodsman A., Jamal S.A., Kaiser S.M. (2010). 2010 clinical practice guidelines for the diagnosis and management of osteoporosis in Canada: Summary. Can. Med. Assoc. J..

[7] Gregson C.L., Armstrong D.J., Bowden J., Cooper C., Edwards J., Gittoes N.J., Harvey N., Kanis J., Leyland S., Low R. (2022). UK clinical guideline for the prevention and treatment of osteoporosis. Arch. Osteoporos..

[8] Kanis J.A., Cooper C., Rizzoli R., Reginster J.Y., Scientific Advisory Board of the European Society for Clinical and Economic Aspects of Osteoporosis (ESCEO) and the Committees of Scientific Advisors and National Societies of the International Osteoporosis Foundation (IOF) (2019). European guidance for the diagnosis and management of osteoporosis in postmenopausal women. Osteoporos. Int..

[9] Kanis J.A. (2010). New osteoporosis guidelines for Canada. Can. Med. Assoc. J..

[10] Cadarette S.M., Carney G., Baek D., Gunraj N., Paterson J.M., Dormuth C.R. (2012). Osteoporosis medication prescribing in British Columbia and Ontario: Impact of public drug coverage. Osteoporos. Int..

[11] Whitaker M., Guo J., Kehoe T., Benson G. (2012). Bisphosphonates for Osteoporosis—Where Do We Go from Here?. N. Engl. J. Med..

[12] Diab D.L., Watts N.B. (2013). Bisphosphonate drug holiday: Who, when and how long. Ther. Adv. Musculoskelet. Dis..

[13] Ro C., Cooper O. (2013). Bisphosphonate drug holiday: Choosing appropriate candidates. Curr. Osteoporos. Rep..

[14] Dennison E.M., Cooper C., Kanis J.A., Bruyère O., Silverman S., McCloskey E., Abrahamsen B., Prieto-Alhambra D., Ferrari S. (2019). Fracture risk following intermission of osteoporosis therapy. Osteoporos. Int..

[15] Khosla S., Hofbauer L.C. (2017). Osteoporosis treatment: Recent developments and ongoing challenges. Lancet Diabetes Endocrinol..

[16] Cosman F., de Beur S.J., LeBoff M.S., Lewiecki E.M., Tanner B., Randall S., Lindsay R. (2014). Clinician’s Guide to Prevention and Treatment of Osteoporosis. Osteoporos. Int..

[17] The Royal Australian College of General Practitioners, Osteoporosis Australia (2017). Osteoporosis Prevention, Diagnosis and Management in Postmenopausal Women and Men over 50 Years of Age. https://www.racgp.org.au/getattachment/2261965f-112a-47e3-b7f9-cecb9dc4fe9f/Osteoporosis-prevention-diagnosis-and-management-in-postmenopausal-women-and-men-over-50-years-of-age.aspx.

[18] Giangregorio L.M., Macintyre N.J., Thabane L., Skidmore C.J., Papaioannou A. (2013). Exercise for improving outcomes after osteoporotic vertebral fracture. Cochrane Database Syst. Rev..

[19] Gibbs J.C., MacIntyre N.J., Ponzano M., Templeton J.A., Thabane L., Papaioannou A., Giangregorio L.M. (2019). Exercise for improving outcomes after osteoporotic vertebral fracture. Cochrane Database Syst. Rev..

[20] WHO Scientific Group on the Prevention and Management of Osteoporosis (2003). Prevention and Management of Osteoporosis: Report of a WHO Scientific Group.

[21] Khosla S. (2003). Surrogates for fracture endpoints in clinical trials. J. Bone Miner. Res..

[22] Bergen D.J.M., Kague E., Hammond C.L. (2019). Zebrafish as an Emerging Model for Osteoporosis: A Primary Testing Platform for Screening New Osteo-Active Compounds. Front. Endocrinol..

[23] Tonelli F., Bek J.W., Besio R., De Clercq A., Leoni L., Salmon P., Coucke P.J., Willaert A., Forlino A. (2020). Zebrafish: A Resourceful Vertebrate Model to Investigate Skeletal Disorders. Front. Endocrinol..

[24] Gilbert M.J., Zerulla T.C., Tierney K.B. (2014). Zebrafish (*Danio rerio*) as a model for the study of aging and exercise: Physical ability and trainability decrease with age. Exp. Gerontol..

[25] Palstra A.P., Tudorache C., Rovira M., Brittijn S.A., Burgerhout E., Van den Thillart G.E., Spaink H.P., Planas J.V. (2010). Establishing zebrafish as a novel exercise model: Swimming economy, swimming-enhanced growth and muscle growth marker gene expression. PLoS ONE.

[26] Lawrence C., Adatto I., Best J., James A., Maloney K. (2012). Generation time of zebrafish (*Danio rerio*) and medakas (*Oryzias latipes*) housed in the same aquaculture facility. Lab. Anim..

[27] Ali S., Champagne D.L., Spaink H.P., Richardson M.K. (2011). Zebrafish embryos and larvae: A new generation of disease models and drug screens. Birth Defects Res. Part C Embryo Today Rev..

[28] Saleem S., Kannan R.R. (2018). Zebrafish: An emerging real-time model system to study Alzheimer’s disease and neurospecific drug discovery. Cell Death Discov..

[29] Berghmans S., Jette C., Langenau D., Hsu K., Stewart R., Look T., Kanki J.P. (2005). Making waves in cancer research: New models in the zebrafish. BioTechniques.

[30] Avdesh A., Chen M., Martin-Iverson M.T., Mondal A., Ong D., Rainey-Smith S., Taddei K., Lardelli M., Groth D.M., Verdile G. (2012). Regular care and maintenance of a zebrafish (*Danio rerio*) laboratory: An introduction. J. Vis. Exp. JoVE.

[31] Nasiadka A., Clark M.D. (2012). Zebrafish breeding in the laboratory environment. ILAR J..

[32] Meng X., Noyes M.B., Zhu L.J., Lawson N.D., Wolfe S.A. (2008). Targeted gene inactivation in zebrafish using engineered zinc-finger nucleases. Nat. Biotechnol..

[33] Doyon Y., McCammon J.M., Miller J.C., Faraji F., Ngo C., Katibah G.E., Amora R., Hocking T.D., Zhang L., Rebar E.J. (2008). Heritable targeted gene disruption in zebrafish using designed zinc-finger nucleases. Nat. Biotechnol..

[34] Huang P., Xiao A., Zhou M., Zhu Z., Lin S., Zhang B. (2011). Heritable gene targeting in zebrafish using customized TALENs. Nat. Biotechnol..

[35] Hwang W.Y., Fu Y., Reyon D., Maeder M.L., Tsai S.Q., Sander J.D., Peterson R.T., Yeh J.R.J., Joung J.K. (2013). Efficient genome editing in zebrafish using a CRISPR-Cas system. Nat. Biotechnol..

[36] Hwang W.Y., Fu Y., Reyon D., Maeder M.L., Kaini P., Sander J.D., Joung J.K., Peterson R.T., Yeh J.R.J. (2013). Heritable and precise zebrafish genome editing using a CRISPR-Cas system. PLoS ONE.

[37] Huo L., Wang L., Yang Z., Li P., Geng D., Xu Y. (2018). Prednisolone induces osteoporosis-like phenotypes via focal adhesion signaling pathway in zebrafish larvae. Biol. Open..

[38] Howe K., Clark M.D., Torroja C.F., Torrance J., Berthelot C., Muffato M., Collins J.E., Humphray S., McLaren K., Matthews L. (2013). The zebrafish reference genome sequence and its relationship to the human genome. Nature.

[39] Dietrich K., Fiedler I.A., Kurzyukova A., López-Delgado A.C., McGowan L.M., Geurtzen K., Hammond C.L., Busse B., Knopf F. (2021). Skeletal Biology and Disease Modeling in Zebrafish. J. Bone Miner. Res..

[40] Barrett R., Chappell C., Quick M., Fleming A. (2006). A rapid, high content, in vivo model of glucocorticoid-induced osteoporosis. Biotechnol. J..

[41] Chen J.R., Lai Y.H., Tsai J.J., Hsiao C.D. (2017). Live Fluorescent Staining Platform for Drug-Screening and Mechanism-Analysis in Zebrafish for Bone Mineralization. Molecules.

[42] Patton E.E., Zon L.I., Langenau D.M. (2021). Zebrafish disease models in drug discovery: From preclinical modelling to clinical trials. Nat. Rev. Drug Discov..

[43] Fernandes Y., Rampersad M., Gerlai A.R. (2015). Impairment of social behaviour persists two years after embryonic alcohol exposure in zebrafish: A model of fetal alcohol spectrum disorders. Behav. Brain Res..

[44] Huang H., Lin H., Lan F., Wu Y., Yang Z., Zhang J. (2018). Application of bone transgenic zebrafish in anti-osteoporosis chemical screening. Anim. Models Exp. Med..

[45] He H., Wang C., Tang Q., Yang F., Xu Y. (2018). Possible mechanisms of prednisolone-induced osteoporosis in zebrafish larva. Biomed. Pharmacother..

[46] Zhang W., Xu J., Qiu J., Xing C., Li X., Leng B., Su Y., Lin J., Lin J., Mei X. (2018). Novel and rapid osteoporosis model established in zebrafish using high iron stress. Biochem. Biophys. Res. Commun..

[47] MacRae C.A., Peterson R.T. (2015). Zebrafish as tools for drug discovery. Nat. Rev. Drug Discov..

[48] Luo Q., Liu S., Xie L., Yu Y., Zhou L., Feng Y., Cai D. (2019). Resveratrol Ameliorates Glucocorticoid-Induced Bone Damage in a Zebrafish Model. Front. Pharmacol..

[49] Spoorendonk K.M., Peterson-Maduro J., Renn J., Trowe T., Kranenbarg S., Winkler C., Schulte-Merker S. (2008). Retinoic acid and Cyp26b1 are critical regulators of osteogenesis in the axial skeleton. Development.

[50] Jun Du S., Frenkel V., Zohar Y., Kindschi G. (2001). Visualizing normal and defective bone development in zebrafish embryos using the fluorescent chromophore calcein. Dev. Biol..

[51] White R.M., Sessa A., Burke C., Bowman T., LeBlanc J., Ceol C., Bourque C., Dovey M., Goessling W., Burns C.E. (2008). Transparent Adult Zebrafish as a Tool for In Vivo Transplantation Analysis. Cell Stem Cell.

[52] Sakata-Haga H., Uchishiba M., Shimada H., Tsukada T., Mitani M., Arikawa T., Shoji H., Hatta T. (2018). A rapid and nondestructive protocol for whole-mount bone staining of small fish and Xenopus. Sci. Rep..

[53] Hur M., Gistelinck C.A., Huber P., Lee J., Thompson M.H., Monstad-Rios A.T., Watson C.J., McMenamin S.K., Willaert A., Parichy D.M. (2017). MicroCT-based phenomics in the zebrafish skeleton reveals virtues of deep phenotyping in a distributed organ system. eLife.

[54] Suniaga S., Rolvien T., Vom Scheidt A., Fiedler I.A., Bale H.A., Huysseune A., Witten P.E., Amling M., Busse B. (2018). Increased mechanical loading through controlled swimming exercise induces bone formation and mineralization in adult zebrafish. Sci. Rep..

[55] Palstra A.P., Rovira M., Rizo-Roca D., Torrella J.R., Spaink H.P., Planas J.V. (2014). Swimming-induced exercise promotes hypertrophy and vascularization of fast skeletal muscle fibres and activation of myogenic and angiogenic transcriptional programs in adult zebrafish. BMC Genom..

[56] McClelland G.B., Craig P.M., Dhekney K., Dipardo S. (2006). Temperature- and exercise-induced gene expression and metabolic enzyme changes in skeletal muscle of adult zebrafish (*Danio rerio*). J. Physiol..

[57] Rovira M., Arrey G., Planas J.V. (2017). Exercise-Induced Hypertrophic and Oxidative Signaling Pathways and Myokine Expression in Fast Muscle of Adult Zebrafish. Front. Physiol..

[58] Usui T., Noble D.W.A., O’Dea R.E., Fangmeier M.L., Lagisz M., Hesselson D., Nakagawa S. (2018). The French press: A repeatable and high-throughput approach to exercising zebrafish (*Danio rerio*). PeerJ.

[59] Huang S.H., Tsao C.W., Fang Y.H. (2020). A Miniature Intermittent-Flow Respirometry System with a 3D-Printed, Palm-Sized Zebrafish Treadmill for Measuring Rest and Activity Metabolic Rates. Sensors.

[60] Bek J.W., De Clercq A., Coucke P.J., Willaert A. (2021). The ZE-Tunnel: An Affordable, Easy-to-Assemble, and User-Friendly Benchtop Zebrafish Swim Tunnel. Zebrafish.

[61] Kilroy E.A., Ignacz A.C., Brann K.L., Schaffer C.E., Varney D., Alrowaished S.S., Silknitter K.J., Miner J.N., Almaghasilah A., Spellen T.L. (2022). Beneficial impacts of neuromuscular electrical stimulation on muscle structure and function in the zebrafish model of Duchenne muscular dystrophy. eLife.

[62] Zebrafish International Resource Center. https://zebrafish.org/home/guide.php.

[63] Hanwell D., Hutchinson S.A., Collymore C., Bruce A.E., Louis R., Ghalami A., Allison W.T., Ekker M., Eames B.F., Childs S. (2016). Restrictions on the Importation of Zebrafish into Canada Associated with Spring Viremia of Carp Virus. Zebrafish.

[64] Song H.D., Sun X.J., Deng M., Zhang G.W., Zhou Y., Wu X.A., Sheng Y., Chen Y., Ruan Z., Jiang C.L. (2004). Hematopoietic gene expression profile in zebrafish kidney marrow. Proc. Natl. Acad. Sci. USA.

[65] Taylor J.S., Braasch I., Frickey T., Meyer A., Van de Peer Y. (2003). Genome duplication, a trait shared by 22,000 species of ray-finned fish. Genome Res..

[66] Schlueter P.J., Royer T., Farah M.H., Laser B., Chan S.J., Steiner D.F., Duan C. (2006). Gene duplication and functional divergence of the zebrafish insulin-like growth factor 1 receptors. FASEB J..

